# Agrupamentos de Fatores de Risco Cardiometabólicos e sua Associação com Aterosclerose e Inflamação Crônica em Adultos e Idosos em Florianópolis, Sul do Brasil

**DOI:** 10.36660/abc.20200230

**Published:** 2021-07-15

**Authors:** Tiago Rodrigues de Lima, Diego Augusto Santos Silva, Maruí Weber Corseuil Giehl, Eleonora D’Orsi, David Alejandro González-Chica

**Affiliations:** 1 Universidade Federal de Santa CatarinaFlorianópolisSCBrasilUniversidade Federal de Santa Catarina, Florianópolis, SC - Brasil; 2 Adelaide Medical SchoolAdelaideSouth AustraliaAustráliaAdelaide Medical School, Adelaide, South Australia - Austrália

**Keywords:** Doenças Cardiovasculares, Adulto, Idoso, Epidemiologia, Síndrome Metabólica, Transtornos Metabólicos dos Lipídeos, Aterosclerose, Inflamação, Espessura Intima Média Carotídea, Proteína C Reativa, Fatores de Risco

## Abstract

**Fundamento:**

O aumento significativo de doenças cardiovasculares em países em desenvolvimento alerta sobre seu impacto em populações carentes.

**Objetivo:**

Identificar a relação de agrupamentos de componentes da síndrome metabólica (SM) com aterosclerose e inflamação crônica em adultos e idosos.

**Métodos:**

Análise transversal usando dados de dois estudos populacionais de tipo coorte realizados em Florianópolis, sul do Brasil (EpiFloripa Adult Cohort Study, n = 862, 39,9±11,5 anos; EpiFloripa Aging Cohort Study, n = 1197, 69,7±7,1 anos). Pressão arterial (PA), circunferência da cintura (CC), e níveis plasmáticos de lipídio e glicose foram analisados como fatores individuais ou como agrupamentos de componentes da SM (como número de componentes presentes em um indivíduo ou como combinações). Os desfechos incluíram espessura intima-media carotídea (EIMC), placas ateroscleróticas, e níveis de proteína C reativa (CRP). Regressão linear múltipla e regressão logística, ajustadas quanto aos fatores de confusão, foram usadas para análise. O nível de significância adotado foi de 5%.

**Resultados:**

Indivíduos com PA e CC elevadas, dislipidemia e hiperglicemia (61,5%) apresentaram maiores valores de EIMC e PCR que aqueles que não apresentaram componentes de SM. CC elevada foi um determinante comum de inflamação sistêmica, ao passo que a coexistência de PA elevada e CC elevada (agrupamentos de dois ou três fatores) associou-se com maior EIMC (β entre +3,2 e +6,1 x 10-2 mm; p < 0,05) e PCR (_EXP_β entre 2,18 e 2,77; p < 0,05).

**Conclusão:**

A coexistência de PA e CC elevadas associou-se com maiores valores de EIMC e níveis de PCR. A obesidade central, isolada ou em combinação com outros fatores de risco, teve efeito sobre a inflamação sistêmica.

## Introdução

As doenças cardiovasculares (DCV) são as principais causas de morte em todo o mundo, responsáveis por um número estimado de 17,8 milhões de mortes em 2017, correspondendo a 330 milhões de anos perdidos e 35,6 milhões de anos vividos com incapacidade.^1,2^ A Organização Mundial da Saúde estima que o número de mortes por DCV atingirá 23,6 milhões até o ano de 2030, principalmente por doenças cardíacas e infarto.^3^ Aproximadamente 75% das DCV são preveníveis, e um controle adequado dos fatores de risco cardiometabólicos (hipertensão, excesso de gordura corporal, hiperglicemia, dislipidemia) é crucial para reduzir morbidade e mortalidade.^3^

O mecanismo fisiopatológico da relação entre DCV e os fatores de risco cardiometabólicos envolve inflamação crônica – altos níveis de proteína C reativa (PCR), interleucina-6, fator de necrose tumoral alfa – bem como alterações micro e macrovasculares.^4,5^ Biomarcadores de inflamação crônica estão diretamente relacionados com gênese da aterosclerose, desenvolvimento de placas instáveis,^6^ e a maioria das DCV.^7^

Segundo a literatura, alguns fatores de risco cardiometabólicos são mais aterogênicos, e embora agrupamentos desses fatores possam coexistir em um mesmo indivíduo, seu efeito combinado sobre a aterosclerose e inflamação crônica foi pouco investigado.^8-10^ Evidências sugerem que o efeito aterogênico dos fatores de risco cardiometabólicos depende de qual combinação está afetando um indivíduo, e que pode ser exacerbado pela coexistência de um estilo de vida não saudável (p.ex. tabagismo, estilo de vida sedentário, hábitos alimentares inadequados).^8-10^ Portanto, identificar agrupamentos de fatores de risco cardiometabólicos com forte efeito aterogênico pode contribuir para o desenvolvimento de estratégias preventivas mais bem direcionadas. Estudos nessa área são particularmente importantes para países de renda baixa e média, uma vez que, em termos absolutos, mortes precoces relacionadas a DCV estão concentradas nesses países.^3,11^ No entanto, a maioria dos estudos sobre esse tópico são conduzidos em lugares de alta renda.^9,10^

Portanto, este estudo tem como objetivo identificar a relação de agrupamentos de fatores de risco cardiometabólicos com aterosclerose e indicadores de inflamação crônica, isto é, espessura intima-media carotídea (EIMC), presença de placa aterosclerótica, e níveis de proteína C-reativa (PCR) na população, utilizando uma amostra composta por adultos e idosos em Florianópolis, sul do Brasil.

## Métodos

Este estudo tem um delineamento transversal baseado em dados de dois estudos populacionais do tipo coorte (EpiFloripa Adult Cohort Study and EpiFloripa Aging Cohort Study). Ambos os estudos foram realizados com residentes de Florianópolis, uma capital localizada no sul do Brasil. A cidade é predominantemente urbana (421.240 habitantes, 59% adultos), com um índice de desenvolvimento humano municipal de 0,847 (o terceiro maior do país) e uma expectativa de vida de 77,3 anos.^12^

**Figura 1:**
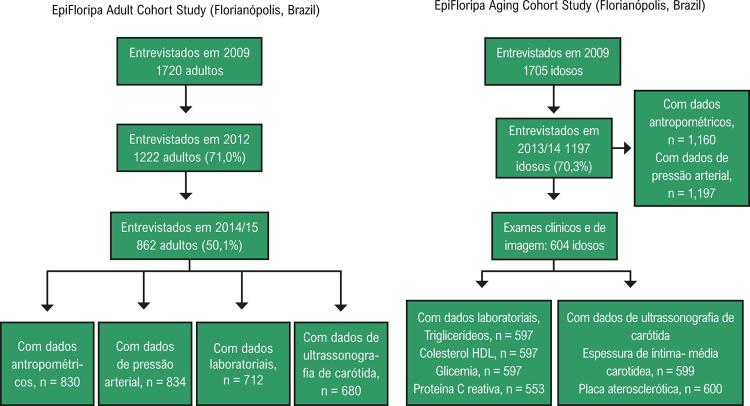
– Fluxograma do EpiFloripa Adult Cohort Study e do EpiFloripa Aging Cohort Study e variáveis usadas no presente estudo.

Detalhes desses dois estudos podem ser encontrados em publicações anteriores.^13-15^ Em resumo, o momento basal do EpiFloripa Adult Cohort Study ocorreu em 2009, quanto 1720 indivíduos com idade entre 20 e 59 anos foram entrevistados em suas residências. A amostragem foi realizada em dois estágios: primeiramente, 10 setores censitários foram selecionados sistematicamente em cada decil da renda familiar (63/420 setores da cidade) e, subsequentemente, 1134/16 755 residências desses setores foram selecionadas sistematicamente. Considerando uma média de 1,78 indivíduos por residência, o processo de amostragem permitiria a identificação de 2016 adultos. Indivíduos com amputação, acamados, hospitalizados, e aqueles com doença mental grave que os impedissem de responder o questionário foram excluídos. Todos os adultos incluídos no momento basal foram rastreados em 2012-2013 e 2014-2015 (segunda e terceira ondas, respectivamente) (Figura 1). Um total de 862 indivíduos foram efetivamente avaliados em 2014-2015 (50,1% da coorte original) nas instalações da Universidade Federal de Santa Catarina (UFSC).

O momento basal do EpiFloripa Aging Cohort Study ocorreu em 2009 (N=1705; faixa etária de 60-104 anos), e a segunda onda ocorreu em 2013/2014. O tamanho da amostra final foi estimado em 1599 indivíduos e uma estratégia de amostragem similar (em dois estágios) foi empregada. Neste caso, oito setores censitários por decil de renda familiar foram selecionados no primeiro estágio, e 60 residências em cada desses setores foram selecionadas no segundo estágio. Uma amostra de 1911 indivíduos seria estimada por esse método de amostragem, considerando uma média de um idoso por cada 2,51 residências. Todos os idosos que viviam nessas residências selecionadas foram considerados elegíveis, exceto idosos institucionalizados. Todos esses indivíduos foram rastreados em 2013-2014, com uma taxa de acompanhamento de 70,3% (n=1197). Desses, 604 aceitaram a participar dos exames clínicos e de imagem, e dos testes de monitoramento.

Os dois estudos foram aprovados pelos comitês de ética da UFSC. Todos os participantes assinaram um termo de consentimento. Em todos os estágios do estudo EpiFloripa Adult Cohort Study e do EpiFloripa Aging Cohort Study, os equipamentos foram calibrados previamente, e os entrevistadores treinados e padronizados quanto às técnicas de medida antropométrica (medindo-se a variabilidade entre e intraobservador).^16^

### Coleta de dados, dados laboratoriais e exame de imagem

Dados antropométricos, laboratoriais e de imagem usados neste artigo foram obtidos da terceira etapa de coleta de dados (terceira onda) do estudo EpiFloripa Adult Cohort Study e da segunda etapa do estudo EpiFloripa Aging Cohort Study. Métodos e equipamentos similares foram usados em ambos os estudos. Medidas antropométricas [circunferência da cintura (CC), altura e massa corporal] foram medidas duas vezes de acordo com as recomendações de Lohman et al.,^16^ e a média das duas medidas foi considerada para o estudo.

A CC foi medida com 1 mm de precisão, na porção mais estreita do tronco usando uma fita métrica inelástica. A altura foi medida utilizando-se um estadiômetro de 1 mm de precisão. A massa corporal foi medida por uma balança digital com precisão de 100 g e capacidade de 150 Kg, calibrada antes do início do estudo.

A pressão arterial foi medida no braço direito do indivíduo sentado, usando um esfigmomanômetro de pulso (Techline® digital read, São Paulo, Brasil), com o braço apoiado ao nível do coração. A pressão arterial foi medida duas vezes, com um intervalo de repouso de pelo menos 15 minutos antes e entre medidas. Quando a diferença entre as medidas foi maior que 20 mmHg para pressão sistólica ou 10 mmHg para pressão diastólica, uma terceira medida foi tomada para substituir o maior valor. A média de duas medidas foi considerada para análise.

As amostras de sangue foram coletadas cedo pela manhã após jejum de pelo menos oito horas. As amostras foram armazenadas e analisadas seguindo padrões técnicos do Laboratório de Análise Clínica (Hospital Universitário, UFSC). Glicemia de jejum e perfil lipídico (triglicerídeos e lipoproteína de alta densidade, HDL) foram determinados pelo teste colorimétrico, e a PCR determinada por turbidimetria.

A ultrassonografia de carótidas foi realizada por um cardiologista, utilizando um equipamento portátil Viamo® (Toshiba Medical System, Tóquio, Japão) com um transdutor linear 5-11MHz. Foram obtidas no mínimo três imagens da EIMC de cada lado (direito e esquerdo), e a imagem de melhor qualidade foi usada nas análises (índice de qualidade >0,50). Medidas da EIMC foram analisadas usando o software M’Ath® (versão 3.1, METRIS Co., Argenteuil, França), que realiza 100 medidas automáticas por centímetro. A média (mm) da EIMC esquerda e direita foi usada nas análises.^17^ A presença de placas ateroscleróticas (carótida comum, bulbo carotídeo, ou ramificações em qualquer dos lados) também foi detectada durante o exame. Placa aterosclerótica foi definida como uma estrutura focal invadindo pelo menos 0,5 mm o lúmen arterial ou apresentando uma espessura de parede ≥50% a espessura da íntima-média circundante, com ou sem calcificações.^18,19^

### Desfechos

Três diferentes desfechos foram considerados neste estudo: 1) EIMC (EIMC média esquerda e direita em milímetros, variável simétrica contínua); 2) presença de placa aterosclerótica (variável binária, sim/não), e 3) níveis de PCR (variável contínua analisada como logaritmo natural, ln, devido à sua assimetria).^20^

### Exposição: agrupamentos de fatores de risco cardiometabólicos

Os critérios aceitos para a definição da síndrome metabólica (SM) foram usados para estabelecer os pontos de corte dos diferentes fatores de risco.^21^ Obesidade central foi definida como CC > 80 cm em mulheres e > 90 cm em homens. Hipertensão foi definida como uma pressão arterial sistólica ≥ 130 mmHg e/ou pressão arterial diastólica ≥ 85 mmHg. Indivíduos que relataram já terem sido diagnosticados com hipertensão por um médico e/ou estavam em uso de drogas anti-hipertensivas também foram classificados como hipertensos. Hiperglicemia foi definida como glicemia de jejum ≥ 100 mg/dL, relato de diagnóstico médico de diabetes mellitus, e/ou uso de hipoglicemiantes.^19^ Os participantes foram considerados dislipidêmicos se apresentassem níveis elevados de triglicerídeos (≥150 mg/dL), níveis baixos de HDL (< 40 mg/dL em homens e < 50 mg/dL em mulheres), ou relatassem uso de hipolipemiantes.^21^

Cada componente da SM foi analisada ou como uma variável independente de exposição ou como um agrupamento. Duas variáveis foram geradas para identificação dos agrupamentos. A primeira variável considerou o número de fatores de risco cardiometabólicos presentes no mesmo indivíduo (0, 1, 2, 3 ou 4 componentes de SM). A segunda combinou as quatro variáveis independentes em 16 combinações possíveis: negativo para todos os riscos, positivo para um fator (quatro combinações possíveis), agrupamento de dois fatores (seis combinações possíveis), agrupamento de três fatores (quatro combinações possíveis) ou todos os fatores de risco cardiometabólicos.

### Variáveis sociodemográficas e de estilo de vida

Dados sociodemográficos e de estilo de vida foram incluídos como possíveis fatores de confusão. A maioria dessas variáveis foram coletadas durante a terceira onda do EpiFloripa Aging Cohort Study (2013/2014): sexo (masculino ou feminino); idade em anos, escolaridade (0-8, 9-11, ≥ 12 anos); renda familiar per capita (incluindo todas as fontes de renda, dividida pelo número de membros da família) distribuída em tercis (1º tercil = <R$ 900,00; 2º tercil= R$ 907,33 a R$ 2122,00; 3º tercil =>R$ 2125,00; 1 USD = R$ 3,14 em in 2015. Tabagismo foi analisado como não fumante, ex-fumante, ou fumante, independentemente da intensidade e da frequência. Um questionário validado foi utilizado para avaliar atividade física em cada coorte.^22,23^ Os adultos foram considerados ativos quando relataram a prática de atividade física de intensidade moderada por 30 minutos ou mais em cinco ou mais dias da semana, ou atividade de alta intensidade por 20 minutos ou mais em três ou mais dias por semana.^24^ Os idosos foram considerados fisicamente ativos quando relataram prática de atividade física por pelo menos 150 minutos por semana. Dados sobre a ingestão energética proveniente do consumo de álcool, gordura saturada, açúcar, fibra e sódio foram obtidos por recordatório 24 horas, e as informações foram extraídas usando procedimentos recomendados na literatura, com ajustes quanto à variabilidade intraindividual e entre indivíduos.^25^ O consumo de gorduras saturadas e fibras foi transformado em *ln* dada sua distribuição assimétrica.

### Análise estatística

Variáveis contínuas simétricas foram descritas em média e desvio padrão, e variáveis assimétricas em mediana e intervalo interquartil (p25-p75). Também foi estimada a média geométrica para PCR. Histogramas e gráficos Q-Q foram usados para verificar a normalidade das variáveis contínuas. As variáveis categóricas foram apresentadas em porcentagens (%). Dependendo da natureza das variáveis investigadas, foram usados o teste do qui-quadrado, teste t para amostras independentes, ou o teste de Mann-Whitney para identificar possíveis diferenças entre membros da coorte avaliados ou não durante a última onda, em comparação ao basal. Apesar de a literatura ter relatado uma importante relação entre mais anos de vida e risco cardiometabólico,^20^ considerando os desfechos do presente estudo (EIMC, placa de carótida e PCR), foram testadas as interações entre todas as variáveis cardiometabólicas (analisadas individualmente ou como agrupamentos) e idade. No entanto, no presente estudo, identificou-se um possível efeito da idade somente sobre PCR e dislipidemia (p=0,61).^26^ Assim, a fim de manter o poder estatístico para testar as associações de interesse neste estudo, as análises foram estratificadas por idade.

Para avaliar os agrupamentos mais prevalentes de fatores de risco metabólico, foi estimada a razão entre a prevalência observada e a esperada (O/E) para cada das 16 possíveis combinações.^27^ A prevalência esperada foi calculada multiplicando-se a probabilidade observada de cada fator de risco, assumindo-se uma ocorrência independente na população. Uma razão O/E > 1,2 foi usada para identificar agrupamentos altamente prevalentes (isto é, mais alta que uma ocorrência aleatória).^27^

Modelos de regressão linear foram usados para testar associações entre os agrupamentos de fatores de risco cardiometabólicos (variáveis independentes) e EIMC e logaritmo natural da PCR (lnPCR). Os resultados foram apresentados como coeficientes de regressão (β) e respectivo erro padrão. Para lnPCR, o β foi transformado em sua forma exponencial (_EXP_β) e interpretado como a porcentagem de aumento nos níveis séricos de PCR em relação à média geométrica daqueles sem nenhum fator de risco cardiometabólicos. Premissas de linearidade das variáveis contínuas, variância constante dos resíduos padronizados, e adequação do ajuste do modelo foram avaliadas plotando-se os resíduos contra os valores ajustados.

Regressão logística foi usada para analisar a presença de placa aterosclerótica como um desfecho, e os resultados foram expressos como razão de changes (*odds ratio*, OR) e respectivos erros padrões. Todas as análises foram ajustadas quanto aos possíveis fatores de confusão (variáveis de estilo de vida e variáveis sociodemográficas),^20^ independentemente do nível de significância estatística na associação com os desfechos.

A análise de dados foi realizada utilizando-se o programa estatístico Stata 13.0 (StataCorp LP, College Station, EUA), considerando os pesos de amostragem, ou seja, a probabilidade de seleção no basal, a probabilidade de localidade durante o acompanhamento, ponderados para a população estimada de Florianópolis por sexo e grupo idade,^12^ e o delineamento de pesquisa de cada estudo. Um valor de p<0,05 foi considerado estatisticamente significativo.

## Resultados

### Comparações entre as coortes

No EpiFloripa Adult Cohort Study, 862 indivíduos foram entrevistados em 2014-15 (50,1% da coorte original) (Tabela 1). Renda familiar e tabagismo foram comparáveis à amostra basal (2009). No EpiFloripa Aging Cohort Study, 1197 idosos foram entrevistados em 2013-2014 (70,3%), que apresentaram dados de sexo, renda familiar e tabagismo comparáveis aos dados da amostra basal (2009).

**Tabela 1:** – Comparação das características basais dos participantes no estudo EpiFloripa Adult Cohort Study em 2009 (n = 1720) versus participantes em 2014/15 (n = 862), e dos participantes do estudo EpiFloripa Aging Cohort Study em 2009 (n = 1705) versus 2013/14 (n = 1197)

	EpiFloripa Adult Cohort Study			EpiFloripa Aging Cohort Study		
	2009 (n=1720)	2014/15 (n=862)		2009 (n=1705)	2013/14 (n=1197)	
Variáveis*	**%**	**%**	**Valor p**	**%**	**%**	**Valor p**
Sexo: masculino	44,3	42,5	0,03^‡^	37,5	36,9	0,36^‡^
Idade em anos: média ± DP	38,1 ± 11,6	39,9 ± 11,5	< 0,01^‡^	70,5 ± 7,9	69,7 ± 7,1	< 0,01^‡^
Renda familiar Per capita (R$)^†^: Mediana [p25-p75]	900 [500-1750]	900 [500-1667]	0,55^§^	767 [380-1600]	800 [400-1667]	0,09^§^
**Tabagismo**						
Nunca fumou	54,7	55,8	0,05^//^	59,6	60,2	0,53^//^
Ex-fumante	26,1	27,7		32,0	31,1	
Fumante	19,2	16,5		8,4	8,7	

*: Considerando as informações obtidas em 2009; ^†^: 1 USD = R$ 1,70 em 2009; ^‡^: teste T; ^§^: teste de Mann-Whitney; ^//^: teste do qui-quadrado.

Dados dos desfechos investigados no presente estudo estavam disponíveis em 1301 participantes de ambos os estudos combinados, e os resultados são descritos a seguir. A EIMC média foi 0,64 mm (± 0.15) e a PCR mediana 1,34 mg/L (p25-p75 0,61, 3,48; média geométrica 2,64 mg/L). Ainda, 27,7% dos participantes apresentavam placas carótidas.

### Prevalência de fatores de risco cardiovascular e avaliação multivariada de risco

A prevalência de obesidade central e níveis elevados de pressão arterial foi de 56,8% e 71.5%, respectivamente; 17,7% apresentaram dislipidemia e 22,4% hiperglicemia (Tabela 2). Combinados, 6,1% da amostra foi positiva para os quatro fatores, e 21,8% não apresentaram componente de SM.

**Tabela 2:** – Resultados ajustados para a associação dos fatores de risco cardiometabólicos com espessura íntima-média carotídea, presença de placa de carótida e níveis de proteína C- reativa em adultos e idosos do EpiFloripa Cohort Study (2014/15) e do EpiFloripa Aging Cohort Study (2013/2014) (n= 1301)

	%	EIMC (mm)	Placa de carótida	lnPCR
		β (EP)^‡^	OR (EP)	_EXP_β (EP)^§^
Fatores de risco individuais*				
Obesidade central (% sim)	56,8	0,6 (0,8)	0,69 (0,14)	1,86 (0,15)^¶^
Hipertensão (% sim)	71,5	2,4 (0,7)^¶^	2,12 (0,51)^¶^	1,09 (0,12)
Dislipidemia (% sim)	17,7	1,9 (1,1)	1,67 (0,43)^¶^	1,12 (0,11)
Hiperglicemia (% sim)	22,4	2,9 (0,9)^¶^	1,21 (0,24)	1,22 (0,10)^¶^
**Número de fatores de risco positivos^†^**				
Nenhum//	21,8	*59,1±8,8*^#^	*27,7%*^#^	*0,88±1,5*^#^
1	27,6	1,6 (0,8)	1,02 (0,35)	1,37 (0,20)
2	27,1	3,0 (1,0)	1,53 (0,64)	2,07 (0,28)
3	17,4	5,5 (1,1)	1,88 (0,78)	2,76 (0,41)
4	6,1	7,5 (2,3)	2,01 (0,96)	2,17 (0,33)

EIMC: espessura intima-media carotídea; lnCRP: logaritmo natural da proteína C reativa; EP: erro padrão; mm: milímetros. * Resultados ajustados quanto a sexo, idade, renda familiar, nível educacional, tabagismo, variáveis nutricionais (consumo de gordura saturada, açúcar, álcool, fibra e sódio), nível de atividade física, e ajustes mútuos entre fatores de risco individuais. † Resultados ajustados quanto a sexo, idade, renda familiar, nível educacional, tabagismo, variáveis nutricionais (consumo de gordura saturada, açúcar, álcool, fibra e sódio), nível de atividade física. ‡ – Resultados apresentados como poder (10^-2^). § – Resultados interpretados como porcentagem de incremento da média geométrica. // – Média ± DP ou prevalência na categoria de referência (“nenhum”). ¶– valor p <0,05 em comparação à categoria de referência. # - valor p para tendência <0,05.

Considerando cada componente da SM como uma variável independente, hipertensão e hiperglicemia foram associadas a uma maior EIMC. Hipertensão e dislipidemia foram associadas com presença de placa aterosclerótica, enquanto obesidade central e dislipidemia foram relacionadas a níveis mais elevados de lnPCR.

A Tabela 2 também mostra que houve uma tendência direta entre o número de fatores de risco cardiometabólicos presentes no mesmo indivíduo e os três desfechos (valor de p para tendência < 0,05 em todos os casos) (Tabela 2).

### Prevalência de combinações dos componentes de SM

A prevalência observada da coexistência dos quatro fatores de risco no mesmo indivíduo foi 6,1%, 410% maior (O/E = 5,1) que o que seria esperado para uma ocorrência aleatória. Para a simultaneidade dos três fatores de risco, todas as combinações com obesidade central apresentaram uma razão O/E de 1,2. A presença simultânea de obesidade central e pressão arterial elevada (20,5%) e a presença isolada de pressão arterial elevada (19,5%) foram as combinações mais frequente dois fatores de um fator de risco, respectivamente. No entanto, mesmo entre estas combinações, a razão O/E foi < 0,1. Finalmente, a prevalência de pacientes sem nenhum dos fatores de risco cardiometabólicos foi 110% mais alta que a prevalência esperada. (Tabela 3)

**Tabela 3:** – Prevalência de combinações de componentes de síndrome metabólica em adultos e idosos no EpiFloripa Cohort Study (2014/15) e EpiFloripa Aging Cohort Study (2013/2014) (n= 1301)

Fator de risco	Obesidade central	Pressão arterial elevada	Dislipidemia	Hiperglicemia	n	Prevalência		
						Observada % (IC95%)	Esperada %	O/E
4	+	+	+	+	112	6,1 (4,9-7,6)	1,2	5,1
3	+	+	+	-	68	5,9 (4,6-7,5)	4,3	1,4
	+	+	-	+	209	10,2 (8,1-12,6)	5,7	1,8
	+	-	+	+	09	0,7 (0,3-1,6)	0,5	1,4
	-	+	+	+	10	0,7 (0,3-1,5)	1,6	0,4
2	+	+	-	-	309	20,5 (17,5-23,8)	19,7	1,0
	+	-	+	-	10	0,8 (0,3-1,8)	1,7	0,5
	+	-	-	+	17	1,1 (0,6-2,1)	2,3	0,5
	-	+	+	-	19	2,4 (1,4-4,4)	5,6	0,4
	-	+	-	+	37	1,8 (1,1-2,9)	7,5	0,2
	-	-	+	+	05	0,4 (0,1-1,0)	0,6	0,7
1	-	-	-	+	17	1,5 (0,8-2,7)	3,0	0,5
	-	-	+	-	11	0,8 (0,4-1,4)	2,2	0,4
	-	+	-	-	202	19,5 (16,2-23,2)	25,9	0,8
	+	-	-	-	70	5,8 (4,4-7,6)	7,8	0,7
0	-	-	-	-	196	21,8 (18,7-25,1)	10,3	2,1

*IC: intervalo de confiança; + presença de fator de risco; - ausência de fator de risco; O: prevalência observada; E: prevalência esperada; O/E: razão entre prevalência observada e prevalência esperada*

### Associações entre agrupamentos de componentes da SM e os desfechos investigados

A Tabela 4 apresenta a associação entre as 16 combinações e fatores de risco cardiometabólicos e os desfechos investigados. Todos os grupos incluindo obesidade central e hipertensão (isto é, agrupamentos de dois, três, ou quatro fatores) mostraram EIMC e lnPCR mais altos que indivíduos sem nenhum fator de risco. Por outro lado, a obesidade central foi um fator comum na determinação de inflamação sistêmica, uma vez que se observou um lnPCR mais alto em todas as combinações que incluísse esse fator de risco. Com exceção da obesidade central, a presença isolada de um fator de risco não foi associada com nenhum dos desfechos investigados. Outros agrupamentos mostraram uma prevalência muito baixa (<1%) que permitisse qualquer conclusão mais robusta. Em contraste, apesar de a razão O/E mais alta foi para a coexistência dos quatro fatores, as associações com os desfechos investigados não foram mais fortes que com os agrupamentos de três componentes. Nenhum dos agrupamentos foi associado com uma frequência mais alta de presença de placa na carótida.

**Tabela 4:** – Associação ajustada de agrupamentos de componentes da síndrome metabólica com Espessura íntima-média carotídea, presença de placa de carótida e níveis de proteína C- reativa em adultos e idosos do EpiFloripa Cohort Study (2014/15) e do EpiFloripa Aging Cohort Study (2013/2014) (n= 1301)

		%	EIMC (mm)	Placa de carótida	lnPCR
	n		β (SE)^‡^	OR (SE)	_EXP_β(SE)^§^
**Todas negativas**	**196**	**21,8**	**59,9±8,8^//^**	**11,6%^ //^**	**0,86±1,5^//^**
**Positiva para um fator de risco**					
Obesidade central	70	5,8	0,4 (1,3)	0,24 (0,13)	2,01 (0,38)^†^
Hipertensão	202	19,5	1,7 (0,9)	1,48 (0,53)	1,19 (0,18)
Dislipidemia	11	0,8	2,7 (1,9)	0,92 (0,85)	1,92 (0,76)
Hiperglicemia	17	1,5	3,7 (2,3)	0,71 (0,66)	1,21 (0,37)
**Agrupamentos de dois componentes **					
Obesidade central + Hipertensão	309	20,5	3,2 (0,9)^†^	1,31 (0,54)	2,18 (0,32)^†^
Obesidade central + Dyslipidemia	10	0,8	-1,4 (1,9)	1,08 (1,04)	3,63 (0,90)^†^
Obesidade central + Hiperglicemia	17	1,1	-1,7 (4,6)	0,69 (0,59)	2,24 (0,88)^†^
Hipertensão + Dyslipidemia	19	2,5	5,3 (4,2)	3,14 (2,44)	1,03 (0,27)
Hipertensão + Hiperglicemia	37	1,8	0,8 (3,4)	1,80 (0,88)	2,23 (0,74)^†^
Dislipidemia + Hiperglicemia	05	0,4	9,4 (4,2)^†^	-	4,20 (1,31)^†^
**Agrupamentos com três componentes **					
Obesidade central + Hipertensão + Dyslipidemia	68	5,9	3,9 (1,5)^†^	2,15 (1,07)	2,77 (0,48)^†^
Obesidade central + Hipertensão + Hiperglicemia	209	10,2	6,1 (1,4)^†^	1,58 (0,70)	2,83 (0,42)†
Obesidade central + Dyslipidemia + Hiperglicemia	09	0,7	5,3 (7,3)	1,36 (1,81)	4,06 (1,44)^†^
Hipertensão + Dyslipidemia + Hiperglicemia	10	0,6	11,1 (4,6)^†^	3,01 (2,78)	1,88 (0,84)
Positivas para os quatro fatores de risco	112	6,1	7,4 (2,3)^†^	1,92 (0,91)	2,21 (0,34)^†^

EIMC: Espessura íntima-média carotídea; lnCRP: logaritmo natural da proteína C reativa; EP: erro padrão; mm: milímetros * Resultados ajustados quanto a sexo, idade, renda familiar, nível educacional, tabagismo, variáveis nutricionais (consumo de gordura saturada, açúcar, álcool, fibra e sódio), e nível de atividade física. † valor p < 0,05 em comparação à categoria de referência, indicando que o desfecho é maior naquele grupo. ‡ – resultados apresentados como poder (10-2). § – Resultados interpretados como porcentagem de incremento da média geométrica. // – Média ± DP ou prevalência na categoria de referência (“todas negativas”).

## Discussão

De acordo com a literatura disponível, este é o primeiro estudo populacional conduzido na América Latina com o objetivo de investigar a relação entre agrupamentos de fatores de risco cardiometabólicos e indicadores de aterosclerose e inflamação crônica. Em concordância com os nossos resultados, estudos similares conduzidos na Islândia, Chipre e Espanha^18,28,29^ identificaram que componentes específicos da SM estão associados a um aumento da EIMC, presença de placa aterosclerótica, e níveis elevados de PCR. Contudo, nenhum desses estudos abordou os efeitos de combinações dos componentes da SM.

De acordo com os nossos achados, EIMC e níveis de PCR aumentaram com o número de fatores de risco de SM presentes no mesmo indivíduo. Resultados similares foram relatados em estudos populacionais transversais com adultos e idosos no Chipre (EIMC)^29^ e Espanha (PCR).^28^ Ainda, um estudo populacional do tipo coorte conduzido na Finlândia,^30^ e outro estudo com funcionários de seis universidades públicas no Brasil^31^ relataram que o efeito “aditivo” dos componentes da SM sobre a EIMC foi maior que o efeito específico de cada componente isolado. Tal fato poderia ser explicado pela coexistência de hábitos ruins^32-34^ ou predisposição genética,^35^ que poderiam facilitar o desenvolvimento desses fatores e aumentar seu potencial aterogênico e inflamatório. Além disso, alguns dos agrupamentos compostos por dois e três fatores foram mais fortemente associados com EIMC e PCR que a coexistência dos quatro fatores de risco para SM.

De acordo com a literatura, o efeito deletério desses agrupamentos sobre o desenvolvimento de DCV resulta da combinação de diversos mecanismos fisiopatológicos, incluindo 1) alta concentração de marcadores inflamatórios locais e sistêmicos como consequência de gordura corporal excessiva; 2) aumento na variação de fluxo e oscilação de tensões dentro do vaso devido à pressão arterial elevada, com consequente disfunção endotelial e rigidez arterial; 3) níveis aumentados de ácidos graxos livres e lipoproteína de baixa densidade (LDL) circulantes, o que implica em maior toxicidade ao endotélio e músculo liso adjacente, e 4) dano à parede do vaso sanguíneo causado por glicosilação de lipoproteínas resultante do aumento nos níveis glicêmicos.^36^ Todos esses fatores promovem a atração e acúmulo de macrófagos, mastócitos e células-T ativados na lesão aterosclerótica em progressão, bem como maior rigidez da artéria e inflamação sistêmica.^36^

Apesar de a literatura destacar que a resistência insulínica e a obesidade exercem um papel central no desenvolvimento de SM e DCV,^18,36,37^ a pressão arterial elevada também foi identificada em nosso estudo como um determinante central de inflamação e aterosclerose. Uma elevada EIMC ou níveis altos de PCR foram encontrados em todas as combinações que incluíram pressão arterial elevada e obesidade abdominal. Do ponto de vista da saúde pública, esses achados são preocupantes, uma vez que um terço dos indivíduos (isto é, a combinação de todos os agrupamentos incluindo esses dois fatores: 18,3% + 6,6% + 5,2% + 4,3% = 34,4% ) estariam em um risco aumentado de DCV devido à maior EIMC.

Além disso, a obesidade abdominal foi um fator de risco comum para inflamação sistêmica em nosso estudo. Resultados similares foram observados em adultos e idosos em Portugal, mostrando a obesidade como o mais importante determinante de inflamação sistêmica ou individualmente ou em combinação com outros fatores de risco cardiometabólicos.^38^ Esse achado reforça a ideia de que a inflamação subclínica crônica em indivíduos com obesidade central contribui para a aterosclerose, independentemente da coexistência de resistência insulínica ou dislipidemia.^6^

Por outro lado, quando avaliados como fatores de risco individuais e não como agrupamentos, a presença de placas ateroscleróticas foi 1,7-2,1 vezes mais provável naqueles com dislipidemia ou hipertensão. Outros estudos populacionais identificaram achados similares.^18,39,40^ Estudos longitudinais não somente mostraram que a pressão sistólica aumentada e dislipidemia são fatores de risco independentes para o desenvolvimento de placas ateroscleróticas,^39,41^ mas a vasodilatação e uso prolongado de hipolipemiantes tem um efeito protetor na sua progressão.^41^

Na análise dos agrupamentos, o número reduzido de indivíduos em alguns e o estudo de um desfecho binário provavelmente afetou o poder do estudo para identificar associações.^42^ Contudo, estudos prévios identificaram uma relação consistente entre obesidade, inflamação e aterosclerose.^43^ Mais estudos incluindo análises longitudinais e amostras maiores são necessários para corroborar tais achados.

Apesar dos pontos fortes do estudo (amostra populacional em um país de renda média, o uso de dados mensurados em vez de apenas autorrelatados, e uso de equipamentos calibrados para aumentar a acurácia dos dados), algumas limitações devem ser destacadas. Primeiro, o delineamento transversal dificulta inferências causais, apesar de estudos longitudinais terem mostrado resultados consistentes.^39,41^Segundo, o número insuficiente de indivíduos em alguns agrupamentos diminuiu o poder estatístico do estudo, principalmente para testar associações com desfechos binários. Terceiro, a porcentagem de perdas de seguimento foi considerável, mas é improvável que tenha causado um viés em nossos resultados, uma vez que as características da amostra estudada foram similares às observadas no basal.

## Conclusão

Em conclusão, nossos resultados mostraram que a coexistência de hipertensão e obesidade central foi associada com EIMC e níveis de PCR aumentados. A obesidade central, isolada ou em combinação com outros fatores de risco, teve efeito sobre a inflamação sistêmica. Ainda, alguns agrupamentos com dois e três componentes mostraram associações mais fortes com EIMC e níveis de PCR em comparação a agrupamentos com quatro componentes. A investigação de componentes da SM como variáveis independentes ou não relacionadas poderia comprometer a identificação de agrupamentos de fatores de risco com maior potencial aterogênico ou inflamatório e, consequentemente, de indivíduos em maior risco para DCV. Esses resultados poderiam ajudar médicos e gestores da saúde pública a definir melhores estratégias para reduzir morbidade e mortalidade associadas com essas condições.
