# Supplementary material for: Agrupamentos de Fatores de Risco Cardiometabólicos e sua Associação com Aterosclerose e Inflamação Crônica em Adultos e Idosos em Florianópolis, Sul do Brasil
Source: Arq Bras Cardiol. 2021 Jul 15;117(1):39–48. [Article in Portuguese] doi: 10.36660/abc.20200230 (PMC8294721; doi:10.36660/abc.20200230)
Supplement: Translation

# Translation: Clusters of Cardiometabolic Risk Factors and Their Association with Atherosclerosis and Chronic Inflammation among Adults and Elderly in Florianópolis, Southern Brazil

**Authors:** Tiago Rodrigues de Lima, Diego Augusto Santos Silva, Maruí Weber Corseuil Giehl, Eleonora D’Orsi, David Alejandro González-Chica

**Affiliations:** 1 BrasilUniversidade Federal de Santa Catarina, Florianópolis, SC - Brasil; 2 AustráliaAdelaide Medical School, Adelaide, South Australia - Austrália

**Keywords:** Cardiovascular Diseases, Adult, Aged, Epidemiology, Metabolic Syndrome, Lipid Metabolism Disorders, C-Reactive Protein, Risk Factors

## Abstract

**Background:**

The significant increase in cardiovascular diseases in developing countries alerts about their impact on underprivileged populations.

**Objective:**

To identify the relationship of clusters of metabolic syndrome (MS) components with atherosclerosis and chronic inflammation among adults and elderly.

**Methods:**

Cross-sectional analysis using data from two population-based cohort studies in Florianópolis, Southern Brazil (EpiFloripa Adult Cohort Study, n = 862, 39.9±11.5 years; EpiFloripa Aging Cohort Study, n = 1197, 69.7±7.1 years). Blood pressure (BP), waist circumference (WC), and lipid and glucose levels were analyzed as individual factors or as clusters (either as the number of components present in an individual or as combinations of components). Outcomes included carotid intima-media thickness (IMT), atherosclerotic plaques, and C-reactive protein (CRP) levels. Multiple linear and logistic regression analyses adjusted for confounding factors were used. The statistical significance adopted was 5%.

**Results:**

Individuals with high BP, elevated WC, dyslipidemia and hyperglycemia (6.1% of the sample) showed higher IMT and CRP than those negatives for all MetS components. Elevated WC was a common determinant of systemic inflammation, while the coexistence of high BP and elevated WC (clusters of two or three factors) was associated with higher IMT (β between +3.2 and +6.1 x 10-2 mm; p value < 0.05) and CRP (_EXP_β between 2.18 and 2.77; p value < 0.05).

**Conclusion:**

The coexistence of high BP and elevated WC was associated with increased IMT and CRP levels, but central obesity affected systemic inflammation either alone or in combination with other risk factors.

## Introduction

Cardiovascular diseases (CVD) are the leading cause of death worldwide, accounting for an estimated 17.8 million deaths in 2017, corresponding to 330 million years of life lost and another 35.6 million years lived with disability.^1,2^ The World Health Organization estimates the number of cardiovascular deaths will reach 23.6 million by 2030, mainly because of heart disease and stroke.^3^ Approximately 75% of CVD are preventable, and an appropriate control of cardiometabolic risk factors (high blood pressure, body fat excess, high blood glucose, dyslipidemia) is crucial to reduce morbidity and mortality.^3^

The pathophysiological mechanism of the relationship between CVD and cardiometabolic risk factors involve chronic inflammation – high levels of C-reactive protein (CRP), interleukin-6, tumor necrosis factor-α – as well as micro and macrovascular changes.^4,5^ Biomarkers of chronic inflammation are directly related to the genesis of atherosclerosis, development of unstable plaques,^6^ and most CVD.^7^

According to the literature, some cardiometabolic risk factors are more atherogenic, and although clusters of these factors may coexist within the same individual, their combined effect on atherosclerosis and chronic inflammation has been barely investigated.^8-10^ The available evidence suggests that the atherogenic effect of cardiometabolic risk factors depends on which combination is affecting an individual and may be exacerbated by the coexistence of unhealthy lifestyle (e.g., smoking, sedentary lifestyle, inadequate eating habits).^8-10^Therefore, identifying clusters of cardiometabolic risk factors with a strong atherogenic effect may contribute to the development of better-targeted preventive strategies. Investigations in this field are particularly relevant for low-and-middle-income countries as, in absolute terms, early deaths related to CVD are concentrated in these countries.^3,11^ However, most studies investigating this topic have been performed in high-income settings.^9,10^

Therefore, this study aims to identify the relationship of clusters of cardiometabolic risk factors with atherosclerosis and indicators of chronic inflammation, *i.e*., carotid intima-media thickness (IMT), presence of atherosclerotic plaque, and C-reactive protein levels (CRP) at a population level, by using a community-based sample of adults and elderly individuals in Florianopolis, Southern Brazil.

## Methods

This study has a cross-sectional design based on data from two population-based cohort studies (EpiFloripa Adult Cohort Study and EpiFloripa Aging Cohort Study). Both studies were conducted with individuals living in Florianópolis, a state capital in Southern Brazil. The city is predominantly urban (421,240 habitants, 59% adults), with a Municipal Human Development Index of 0.847 (third largest in Brazil) and life expectancy of 77.3 years.^12^

Details of both cohort studies can be found in previous publications.^13-15^ In summary, the baseline of the EpiFloripa Adult Cohort Study occurred in 2009, when 1,720 individuals aged 20-59 years were interviewed at home. Sampling was performed in two stages: initially 10 census tracts were systematically selected in each decile of family income (63/420 sectors in the city), and subsequently, 1,134/16,755 households in these sectors were also systematically selected. Considering an average of 1.78 adults per household, the sampling process would allow identifying 2,016 adults. Individuals who were amputated or bedridden, hospitalized, or had any severe mental illness that prevented them from responding to the questionnaire were excluded. All adults included in the baseline were traced in 2012-2013 and 2014-2015 (second and third waves, respectively) (Figure 1). A total of 862 individuals were effectively evaluated in 2014-2015 (50.1% of the original cohort) at the premises of the Federal University of Santa Catarina (UFSC).

**Figure 1:** – Flowchart of the EpiFloripa Adult Cohort Study and EpiFloripa Aging Cohort Study and variables used in the present study.

The baseline of the EpiFloripa Aging Cohort Study was conducted in 2009 (N=1,705; age range 60-104 years), and a second wave occurred in 2013/2014. The final sample size at baseline was estimated as 1,599 subjects and a similar two-stage cluster sampling strategy was employed. In this case, eight census tracts per decile of family income were selected in the first stage and 60 households in each of these sectors were selected during the second stage. This sampling method would yield a sample estimate of 1911, considering a mean of one elderly for every 2.51 households. All elderly people residing in the selected households were considered eligible for the study, except institutionalized ones. All these individuals were traced in 2013-2014, with a follow-up rate of 70.3% (n=1,197). Of these, 604 participants accepted to participate in clinical and imaging examinations and monitoring tests.

Ethics approvals for both studies were obtained from the Human Research Ethics Committees of the UFSC. All participants signed a consent form. In all cohort waves of the EpiFloripa Adult Cohort Study and the EpiFloripa Aging Cohort Study, equipment was previously calibrated, and interviewers were trained and standardized in the anthropometric measurement techniques (by inter- and intra-observer variability).^16^

### Data collection, laboratory data, and imaging test

Anthropometric, laboratory and image data used in this article belong to the third wave of the EpiFloripa Adult Cohort Study and the second wave of the EpiFloripa Aging Cohort Study. Similar methods and equipment were used in both studies. Anthropometric measures (waist circumference [WC], height and body mass) were measured twice according to the recommendations of Lohman et al.,^16^ and the mean of two measurements was considered for the study.

WC was measured to the nearest 1mm at the narrowest part of the trunk using an inextensible anthropometric tape, and height was measured to the nearest 1 mm using a stadiometer. Body mass was measured to the nearest 0,1 Kg using a digital scale (150 Kg capacity), calibrated before the study.

Blood pressure was measured in the sitting position, on the right arm using a pulse sphygmomanometer (Techline® digital read, São Paulo, Brazil), with the cuff at heart level. Blood pressure levels were measured twice, with a rest time of at least 15 minutes before and between measurements. When the difference between measurements was greater than 20 mmHg for systolic blood pressure or 10 mmHg for diastolic blood pressure, a third measurement was performed to replace the highest value. The mean of measurements was considered for analysis.

Blood samples were collected early in the morning after fasting for at least eight hours. Samples were stored and analyzed according to standard techniques of the Laboratory of Clinical Analysis (University Hospital, UFSC). Fasting blood glucose and lipid profile (triglycerides and high-density lipoproteins - HDL) were determined by colorimetric test, while CRP was determined by turbidimetry.

Carotid ultrasound was performed by a cardiologist using a portable ultrasound equipment Viamo™ (Toshiba Medical Systems, Tokyo, Japan) using a 5-11 MHz linear transducer. At least three IMT images of the common carotid artery were obtained on each side (right and left) and used the one with the best quality (quality index > 0.50). Carotid IMT measurements were analyzed using the M’Ath® software (version 3.1, METRIS Co., Argenteuil, France), which performs 100 automated measures per centimeter. The mean (in mm) of the left and right IMT was used in the analyses.^17^ The presence of atherosclerotic plaques (common carotid, carotid bulb, or ramifications in any side) was also identified during the imaging examination. Atherosclerotic plaque was defined as a focal structure encroaching at least 0.5 mm into the arterial lumen or having an arterial wall thickness ≥ 50% the surrounding IMT, with or without calcifications.^18,19^

### Outcomes

Three different outcomes were considered for this study: 1) carotid IMT (mean left and right IMT in millimeters, continuous symmetrical variable); 2) the presence of atherosclerotic plaque (binary variable, yes/no), and; 3) CRP levels (continuous variable analyzed as natural logarithm, ln, due to its asymmetry).^20^

### Exposure: clusters of cardiometabolic risk factors

The joint criteria for definition of metabolic syndrome (MS) were used to establish the cutoff points of the different risk factors.^21^ Central obesity was defined as a WC > 80.0 cm in women and > 90.0 cm in men. High blood pressure was defined as a systolic blood pressure ≥ 130 mmHg and/or diastolic blood pressure ≥ 85 mmHg. Individuals who reported being diagnosed with hypertension by a physician and/or were taking antihypertensive drugs were also classified as hypertensive. Hyperglycemia was defined as a fasting blood glucose ≥ 100 mg/dl, self-report of a medical diagnosis of diabetes mellitus, and/or making use of antidiabetic medications.^19^ Participants were considered to have dyslipidemia when they had elevated triglycerides (≥ 150 mg/dL), low HDL (< 40 mg/dl in men and < 50 mg/dl in females), or reported use of antilipemic drugs.^21^

Each MS component was analyzed either as an independent exposure variable or as a cluster. Two different variables were generated to identify clusters. The first variable considered the number of positive cardiometabolic risk factors within the same individual (0, 1, 2, 3 or 4 MetS components). The second one combined the four independent variables into 16 possible combinations: negative for all risk factors, positive for only one factor (four possible combinations), two-factor cluster (six possible combinations), three-factor cluster (four possible combinations) or positive for all cardiometabolic risk factors.

### Sociodemographic and lifestyle variables

Sociodemographic and lifestyle data were included as possible confounding factors. Most of these variables were collected during the third wave of the EpiFloripa Adult Cohort Study (2014/2015) and second wave of the EpiFloripa Aging Cohort Study (2013/2014): sex (male or female); age in years; education attainment (0-8, 9-11, ≥12 years of schooling); *per capita* family income (including all sources of income and divided by the number of family members) distributed in tertiles (1^st^tertile = <R$ 900.00; 2^nd^tertile = R$ 907.33 to R$ 2,122.00; 3^rd^tertile =>R$ 2,125.00 ; 1 USD = R$ 3.14 in 2015). Smoking was analyzed as never smoked, former smoker or smoker, regardless of intensity and frequency. A validated questionnaire was used to evaluate physical activity in each cohort.^22,23^ The adults were considered active when they reported ≥ 30 minutes of moderate-intensity physical activity on five or more days per week or vigorous-intensity activities for at least 20 minutes on three or more days per week.^24^ Older adults were considered physically active when performed ≥ 150 minutes of physical activity/week. Data on energy intake from alcohol, saturated fat, sugar, fiber and sodium intake were obtained by two 24-hour food recalls, and information was extracted using procedures recommended in the literature, with adjustments for intra- and inter-individual variability.^25^ Consumption of saturated fats and fiber were transformed into *ln* due to their asymmetric distribution.

### Statistical analysis

Symmetric, continuous variables were described as mean and standard deviation, and asymmetric variables as median and interquartile range (p25-p75). Geometric mean was also estimated for CRP. Histograms and Q-Q plots were used to verify the normality of continuous variables. Categorical variables were presented as percentages (%). Depending on the nature of the investigated variables, the chi-square test, t-test for independent samples, or the Mann-Whitney test was used to identify possible differences between cohort members evaluated (or not) in the last wave, as compared to the baseline. Although the literature has reported an important relationship between increased years of life and cardiometabolic risk,^20^ considering the outcomes of the present study (IMT, carotid plaque and CRP), interactions between all cardiometabolic risk variables (analyzed individually or as clusters) with age were tested. However, in the present study, a possible effect of age was detected only on CRP and dyslipidemia (p = 0.61).^26^ Thus, to maintain statistical power to test the associations of interest in this study, the analyses were not stratified by age.

To evaluate the most prevalent clusters of cardiometabolic risk factors, the ratio between the observed and expected prevalence (O/E ratio) for each of the 16 possible combinations was estimated.^27^ The expected prevalence was calculated by multiplying the observed probability of each risk factor, assuming that they occur independently in the population. An O/E ratio > 1.2 was used to identify highly prevalent clusters (i.e., higher than a random occurrence).^27^

Linear regression models were used to test associations between the clusters of cardiometabolic risk factors (independent variables) and IMT and natural logarithm of CRP (lnCRP). Results were presented as regression coefficients (β) with the respective standard error (SE). For lnCRP, β was transformed into the exponential form (_EXP_β) and was interpreted as the percentage increase in serum CRP levels in relation to the geometric mean of those without any cardiometabolic risk factor. Assumptions of linearity for continuous variables, constant variance of standardized residuals, and goodness of fit of the model were assessed by plotting residuals against fitted values.

Logistic regression was used to analyze the presence of atherosclerotic plaque as an outcome, and results were expressed as odds ratio (OR) with their respective SE. All analyses were adjusted for all possible confounders (sociodemographic and lifestyle variables),^20^ regardless of their level of statistical significance in the association with the outcomes.

Data analysis was conducted in the statistical software Stata 13.0 (StataCorp LP, College Station, USA), considering sampling weights, i.e., probability of selection at baseline and probability of location during follow-up, re-weighted to the estimated population of Florianópolis by sex and age group^12^ and the survey design in each study. (A p value < 0.05 denoted statistical significance.

## Results

### Comparisons between cohorts

In the EpiFloripa Adult Cohort Study, 862 individuals were interviewed in 2014-15 (50.1% of the original cohort) Table 1). Family income and smoking status of participants were comparable with those of the baseline sample (2009). In the EpiFloripa Aging Cohort Study, 1,197 elderly were interviewed in 2013/14 (70.3%), who showed comparable data of sex, family income and smoking status to the baseline sample (2009).

**Table 1:** – Comparison of baseline characteristics of participants in the EpiFloripa Adult Cohort Study in 2009 (n = 1720) versus participants recruited in 2014/15 (n = 862), and participants in the EpiFloripa Aging Cohort Study in 2009 (n = 1705) versus 2013/14 (n = 1197)

	EpiFloripa Adult Cohort Study			EpiFloripa Aging Cohort Study		
	2009 (n=1,720)	2014/15 (n=862)		2009 (n=1,705)	2013/14 (n=1197)	
Variables*	**%**	**%**	**p value**	**%**	**%**	**p value**
Sex: male	44.3	42.5	0.03^‡^	37.5	36.9	0.36^‡^
Age in years: mean ± SD	38.1 ± 11.6	39.9 ± 11.5	< 0.01^‡^	70.5 ± 7.9	69.7 ± 7.1	< 0.01^‡^
Per capita Family income (R$)^†^: Median [p25-p75]	900 [500-1750]	900 [500-1667]	0.55^§^	767 [380-1600]	800 [400-1667]	0.09^§^
**Smoking**						
Never smoked	54.7	55.8	0.05^//^	59.6	60.2	0.53^//^
Former smoker	26.1	27.7		32.0	31.1	
Smoker	19.2	16.5		8.4	8.7	

*: Considering information obtained in 2009; ^†^: 1 USD = R$ 1.70 in 2009; ^‡^: T- test; ^§^: Mann-Whitney test; ^//^: Chi-square test.

Data of the outcomes of the present study were available in 1,301 participants in both studies combined, and results are described below. The mean IMT was 0.64 mm (± 0.15) and the median CRP was 1.34 mg/L (p25-p75 0.61, 3.48; geometric mean 2.64 mg/L). Also, 27.7% of participants had carotid plaques.

### Prevalence of cardiovascular risk factors and multivariate risk assessment

The prevalence of central obesity and elevated blood pressure levels was 56.8% and 71.5%, respectively; 17.7% of participants had dyslipidemia and 22.4% hyperglycemia (Table 2). Combined, 6.1% of the sample was positive for all four factors, while 21.8% had no MS component.

**Table 2:** – Results adjusted for the association of cardiometabolic risk factors with the carotid intima-media thickness, presence of carotid plaque and C-reactive protein levels among adults and older adults in the EpiFloripa Cohort Study (2014/15) and EpiFloripa Aging Cohort Study (2013/2014) (n= 1,301)

	%	IMT (mm)	Carotid plaque	lnCRP
		β (SE)^‡^	OR (SE)	_**EXP**_β (SE)^§^
Individual risk factors*				
Central obesity (% yes)	56.8	0.6 (0.8)	0.69 (0.14)	1.86 (0.15)^¶^
Elevated blood pressure (% yes)	71.5	2.4 (0.7)^¶^	2.12 (0.51)^¶^	1.09 (0.12)
Dyslipidemia (% yes)	17.7	1.9 (1.1)	1.67 (0.43)^¶^	1.12 (0.11)
Hyperglycemia (% yes)	22.4	2.9 (0.9)^¶^	1.21 (0.24)	1.22 (0.10)^¶^
**Number of positive risk factors^†^**				
None//	21.8	*59.1±8.8*^#^	*27.7%*^#^	*0.88±1.5*^#^
1	27.6	1.6 (0.8)	1.02 (0.35)	1.37 (0.20)
2	27.1	3.0 (1.0)	1.53 (0.64)	2.07 (0.28)
3	17.4	5.5 (1.1)	1.88 (0.78)	2.76 (0.41)
4	6.1	7.5 (2.3)	2.01 (0.96)	2.17 (0.33)

IMT: carotid intima-media thickness; lnCRP: natural logarithm of C-reactive protein; SE: standard error; mm: millimetres. * – Results adjusted for sex, age, family income, educational level, smoking, nutritional variables (intake of saturated fat, sugar, alcohol, fibre, and sodium), physical activity level, and mutual adjustment between individual risk factors. † Results adjusted for sex, age, family income, educational level, smoking, nutritional variables (intake of saturated fat, sugar, alcohol, fibre, and sodium), and physical activity level ‡ – Results presented as power (10^-2^). § – Results interpreted as increment percentage of geometric mean. // – Mean ± SD or prevalence in the reference category (“none”). ¶– p-value <0.05 in comparison with the reference category. # - p-value for trend <0.05.

Considering each MS component as an independent variable, high blood pressure and hyperglycaemia were associated with a higher IMT. High blood pressure and dyslipidemia were associated with the presence of atherosclerotic plaque, whereas central obesity and dyslipidemia were related to higher lnCRP levels.

Table 2 also shows there was a direct trend between the number of cardiometabolic risk factors present in the same individual and the three outcomes (p-value for trend < 0.05 in all cases) (Table 2).

### Prevalence of combinations of MS components

The observed prevalence for the coexistence of the four risk factors in the same individual was 6.1%, 410% higher (O/E ratio = 5.1) than it would be expected at random. For the simultaneity of three risk factors, all combinations with central obesity presented an O/E ratio > 1.2. The simultaneous presence of central obesity and elevated blood pressure (20.5%) and the isolated presence of elevated blood pressure (19.5%) were the most frequent combinations of two and one risk factors, respectively. However, even among these combinations, the O/E ratio was < 1.0. Finally, the observed prevalence of patients with none of the cardiometabolic risk factors was 110% higher than the expected prevalence. (Table 3).

**Table 3:** – Prevalence of combinations of metabolic syndrome components among adults and older adults in the EpiFloripa Cohort Study (2014/15) and EpiFloripa Aging Cohort Study (2013/2014) (n= 1,301)

Risk Factor	Central obesity	Elevated blood pressure	Dyslipidemia	Hyperglycemia	n	Prevalence		
						Observed % (95% CI)	Expected %	O/E
4	+	+	+	+	112	6.1 (4.9-7.6)	1.2	5.1
3	+	+	+	-	68	5.9 (4.6-7.5)	4.3	1.4
	+	+	-	+	209	10.2 (8.1-12.6)	5.7	1.8
	+	-	+	+	09	0.7 (0.3-1.6)	0.5	1.4
	-	+	+	+	10	0.7 (0.3-1.5)	1.6	0.4
2	+	+	-	-	309	20.5 (17.5-23.8)	19.7	1.0
	+	-	+	-	10	0.8 (0.3-1.8)	1.7	0.5
	+	-	-	+	17	1.1 (0.6-2.1)	2.3	0.5
	-	+	+	-	19	2.4 (1.4-4.4)	5.6	0.4
	-	+	-	+	37	1.8 (1.1-2.9)	7.5	0.2
	-	-	+	+	05	0.4 (0.1-1.0)	0.6	0.7
1	-	-	-	+	17	1.5 (0.8-2.7)	3.0	0.5
	-	-	+	-	11	0.8 (0.4-1.4)	2.2	0.4
	-	+	-	-	202	19.5 (16.2-23.2)	25.9	0.8
	+	-	-	-	70	5.8 (4.4-7.6)	7.8	0.7
0	-	-	-	-	196	21.8 (18.7-25.1)	10.3	2.1

*CI: confidence interval; + presence of risk factor; - absence of risk factor; O: observed prevalence; E: expected prevalence; O/E: ratio between observed and expected prevalence.*

### Associations between clusters of MS components and the investigated outcomes

Table 4 shows the association between the 16 combinations of cardiometabolic risk factors and the investigated outcomes. All clusters including central obesity and high blood pressure (*i.e*. clusters of two, three, or four factors) showed higher IMT and lnCRP than individuals without any risk factor. On the other hand, central obesity was a common factor in the determination of systemic inflammation, since a higher lnCRP was observed in all combinations including that risk factor. Except for central obesity, the isolated presence of one risk factor was not associated with any of the investigated outcomes. Other clusters showed a low prevalence (< 1%) to allow robust conclusions. Conversely, although the highest O/E was for the coexistence of the four risk factors, the associations with the investigated outcomes were not stronger than the three-component clusters. None of the clusters was associated with a higher frequency of carotid plaque.

**Table 4:** – Adjusted association* of clusters of metabolic syndrome components with carotid intima-media thickness, presence of carotid plaque and C-reactive protein levels among adults and older adults in the EpiFloripa Cohort Study (2014/15) and EpiFloripa Aging Cohort Study (2013/2014) (n= 1301)

		%	IMT (mm)	Carotid plaque	lnCRP
	n		β (SE)^‡^	OR (SE)	_EXP_β(SE)^§^
**All negative**	**196**	**21.8**	**59.9±8.8^//^**	**11.6% ^//^**	**0.86±1.5^//^**
**Positive for one risk factor only**					
Central obesity	70	5.8	0.4 (1.3)	0.24 (0.13)	2.01 (0.38)^†^
Elevated blood pressure	202	19.5	1.7 (0.9)	1.48 (0.53)	1.19 (0.18)
Dyslipidemia	11	0.8	2.7 (1.9)	0.92 (0.85)	1.92 (0.76)
Hyperglycaemia	17	1.5	3.7 (2.3)	0.71 (0.66)	1.21 (0.37)
**Two-components clusters**					
Central obesity + elevated blood pressure	309	20.5	3.2 (0.9)^†^	1.31 (0.54)	2.18 (0.32)^†^
Central obesity + Dyslipidemia	10	0.8	-1.4 (1.9)	1.08 (1.04)	3.63 (0.90)^†^
Central obesity + Hyperglycemia	17	1.1	-1.7 (4.6)	0.69 (0.59)	2.24 (0.88)^†^
Elevated blood pressure + Dyslipidemia	19	2.5	5.3 (4.2)	3.14 (2.44)	1.03 (0.27)
Elevated blood pressure + Hyperglycemia	37	1.8	0.8 (3.4)	1.80 (0.88)	2.23 (0.74)^†^
Dyslipidemia + Hyperglycemia	05	0.4	9.4 (4.2)^†^	-	4.20 (1.31)^†^
**Three-components clusters**					
Central obesity + Elevated blood pressure + Dyslipidemia	68	5.9	3.9 (1.5)^†^	2.15 (1.07)	2.77 (0.48)^†^
Central obesity + Elevated blood pressure + Hyperglycemia	209	10.2	6.1 (1.4)^†^	1.58 (0.70)	2.83 (0.42)†
Central obesity + Dyslipidemia + Hyperglycemia	09	0.7	5.3 (7.3)	1.36 (1.81)	4.06 (1.44)^†^
Blood pressure + Dyslipidemia + Hyperglycemia	10	0.6	11.1 (4.6)^†^	3.01 (2.78)	1.88 (0.84)
Positives for the four risk factors	112	6.1	7.4 (2.3)^†^	1.92 (0.91)	2.21 (0.34)^†^

IMT: carotid intima-media thickness; CRP: natural logarithm of C-reactive protein; SE: standard error; mm: millimeters. *Results adjusted for sex, age, family income, educational level, smoking, nutritional variables (intake of saturated fat, sugar, fibre, and sodium) and physical activity level. † – p-value< 0.05 in comparison with the reference category, indicating a higher outcome in that group. ‡ – results presented as power (10-2). § – Results should be interpreted as increment percentage of geometric mean. // – Mean ± SD or prevalence in the reference category (“all negative”).

## Discussion

According to the available literature, this is the first population-based study conducted in Latin America aiming to investigate the relationship between clusters of cardiometabolic risk factors and indicators of atherosclerosis and chronic inflammation. In agreement with our results, similar studies conducted in Iceland, Cyprus and Spain^18,28,29^ identified that specific MS components are associated with increased carotid IMT, presence of atherosclerotic plaque, and increased CRP levels. However, none of these studies addressed the effects of combinations of MS components.

According to our findings, IMT and CRP levels increased with the number of MS risk factors present in the same individual. Similar findings were reported in cross-sectional population-based studies investigating adults and older people in Cyprus (IMT)^29^ and Spain (CRP).^28^ Furthermore, a population-based cohort study carried out in Finland^30^ and another study with employees from six public universities in Brazil^31^ reported that the “additive” effect of MS components on carotid IMT was stronger than the effect of each single component. This may be due to the coexistence of harmful habits^32-34^ or genetic predisposition^35^ that could facilitate the development of these factors and increase their atherogenic and inflammatory potential. Moreover, some of the two and three-component clusters had a stronger association with IMT and CRP than the coexistence of the four MS risk factors.

According to the literature, the deleterious effect of these clusters on the development of CVD results of the combination of diverse pathophysiological mechanisms, including 1) high concentration of local and systemic inflammatory markers as a consequence of body fat excess; 2) increased flow variation and oscillation of tensions within the vessel due to high blood pressure, with consequent endothelial dysfunction and arterial stiffness; 3) elevated circulating level of free fatty acids and low-density lipoprotein (LDL) cholesterol, which implies in increased toxicity to the endothelium and adjacent smooth muscle, and 4) blood vessel wall injury caused by lipoprotein glycation resulting from high blood glucose levels.^36^ All these factors promote the attraction and accumulation of activated macrophages, mast cells and T-cells in the growing atherosclerotic lesion, as well as greater arterial stiffness and systemic inflammation.^36^

Although the literature highlights that insulin resistance and obesity play a central role in the development of MS and CVD,^18,36,37^ high blood pressure was also found in our study as a central determinant of inflammation and atherosclerosis. Higher IMT or CRP levels were observed in all combinations that included high blood pressure and abdominal obesity. From a public health perspective, these findings are worrisome as one-third of individuals (*i.e*., the combination of all clusters including these two factors: 18.3% + 6.6% + 5.2% + 4.3% = 34.4%) would be at increased risk of CVD due to higher IMT.

In addition, abdominal obesity was a common risk factor for systemic inflammation in this study. Similar results were observed among adults and elderly in Portugal, abdominal obesity was the most important determinant of systemic inflammation either individually or in combination with other cardiometabolic risk factors.^38^ This finding reinforces the idea that chronic subclinical inflammation among individuals with central obesity contributes to atherosclerosis, regardless of the coexistence of insulin resistance or dyslipidemia.^6^

On the other hand, when assessed as individual risk factors rather than as clusters, the presence of atherosclerotic plaque was 1.7-2.1 more likely among those with dyslipidemia or high blood pressure. Other population-based studies have identified similar findings.^18,39,40^ Longitudinal studies not only showed that increased systolic pressure and dyslipidemia are independent risk factors for the development of atherosclerotic plaque,^39,41^ but vasodilation and prolonged use of lipid-lowering drugs have a protective effect on their progression.^41^

In cluster analysis, the reduced number of individuals in some clusters and the investigation of a binary outcome probably affected the power of the study to identify associations.^42^ However, previous studies have identified a consistent relationship between obesity, inflammation, and atherosclerosis.^43^ Further research including longitudinal analyses and larger samples is necessary to corroborate these findings.

Despite the strengths of the study (population-based sample from a middle-income country, the use of measured data and not only self-reported, and calibrated equipment to increase data accuracy), some limitations need to be highlighted. First, the cross-sectional design limits causal inferences, although longitudinal studies have shown consistent results.^39,41^ Second, the insufficient number of individuals in some clusters reduced the statistical power of the study, especially for testing the associations with binary outcomes. Third, despite the considerable percentage losses to follow-up, it is unlikely that it biased our results, as the characteristics of the investigated sample were similar to those at the baseline.

## Conclusion

In conclusion, our findings showed that the coexistence of elevated blood pressure and central obesity was associated with increased IMT and CRP levels. Central obesity affected systemic inflammation either alone or in combination with other risk factors. Moreover, some two- and three-component clusters showed stronger associations with IMT and CRP levels compared with four-component clusters. The investigation of MS components as independent and unrelated variables could undermine the identification of risk factor cluster with greater atherogenic or inflammatory potential and, consequently, of individuals at a higher risk of CVD. These findings may help clinicians and public health policymakers to define better strategies to reduce morbidity and mortality associated with these conditions.
